# *In Vivo* Ciprofloxacin Release from Hydroxyapatite-Based Bone Implants in Rabbit Tibia: A Preliminary Study

**DOI:** 10.5402/2011/420549

**Published:** 2011-12-07

**Authors:** Amit Kumar Nayak, Ajoy Bhattacharyya, Kalyan Kumar Sen

**Affiliations:** Department of Pharmaceutics, Gupta College of Technological Sciences, Asansol 713301, India

## Abstract

The present study deals with the preliminary evaluation of *in vivo* ciprofloxacin release from HAp-ciprofloxacin bone implants in rabbit tibia. The HAp-ciprofloxacin implants (2 × 2.5 mm) were prepared using various HAp-ciprofloxacin composites synthesized by precipitation technique and 1.5% w/v guar gum as a binder. 5 implants were implanted in artificial cortical bone window at the right proximal tibia of each rabbit. After 3- and 6-day intervals, the rabbits were euthanized. Bone marrow and serum concentrations of ciprofloxacin were determined from the harvested tibia using HPLC method. The results displayed the availability of elevated local antibiotic concentration at the implanted site with the limitation of systemic antibiotic exposure, which can be useful to minimize the risk of systemic toxicity-related side effects. This study is the preliminary investigation of the suitability of *in vivo* ciprofloxacin release from HAp-ciprofloxacin bone implants in rabbit tibia, after implantation, and these implants can be useful for the treatment of bacterial bone infections like osteomyelitis.

## 1. Introduction

Prolonged administration of systemic antibiotics is usually required to treat bacterial bone infections. But the conventional prolonged systemic antibiotic therapy is a insufficient method for achieving high local antibiotic concentration at the diseased bone site as bones are poorly perfused organ [1]. High systemic antibiotics doses to facilitate sufficient tissue penetration are not preferable because of possible toxic systemic side effects. But local sustained antibiotic release implantable delivery may offer considerable advantages over the traditional therapy by producing effective local antibiotic concentration at diseased site with limitation of systemic antibiotic exposure maintaining low systemic side effects [2]. 

Hydroxyapatite (HAp, [Ca_10_(PO_4_)_6_(OH)_2_]) is a bioceramic used in orthopedics and dentistry applications due to its close similarities with mineral component of bone and teeth [3]. Because of its biocompatibility and bone-bonding property, HAp has been used as a safe matrix in the bone drug delivery [4, 5]. It also offers high binding affinity for a variety of pharmacological substances such as antibiotics, hormones, and steroids [6–8]. This has opened the potential for using HAp to deliver various pharmacological substances in many clinical applications. Synthetic HAp is known to be similar to naturally occurring HAp on the basis of crystallographic and chemical studies [3]. Different techniques have been employed to produce HAp synthetically [3, 9]. 

Fluoroquinolones are considered as the drug of choice for bone infections [10]. Among them, ciprofloxacin is widely used in bacterial bone infections because of its favorable penetration and antibactericidal activity on all the probable pathogens [11]. In the literature, a few ciprofloxacin-loaded HAp-based implantable systems developed using precipitation technique are reported [11–14]. In our previous investigation, we have developed HAp-based bone implants using synthesized HAp-ciprofloxacin composites by precipitation technique and 2% w/v aqueous solution of guar gum as binder. The *in vitro* performance of HAp-ciprofloxacin bone implants (2 × 2.5 mm) was evaluated, and the result showed a good promise of their use [14]. In the present investigation, the *in vivo* ciprofloxacin release from HAp-ciprofloxacin bone implants implanted in rabbit tibia was evaluated.

## 2. Materials and Methods

### 2.1. Materials

Ciprofloxacin HCl was a gift from Dr. Reddy's Laboratories (India). Calcium hydroxide and orthophosphoric acid were procured from Qualigens Fine Chemicals, India. Triethanolamine, acetonitrile for chromatography, and water for chromatography were purchased from Merck Specialties Pvt. Ltd., India. Guar gum was purchased from HiMedia Laboratories, India. All other chemicals were of analytical grade.

### 2.2. Synthesis of HAp-Ciprofloxacin Composites

The HAp-ciprofloxacin composites were synthesized by precipitation technique [14]. In brief, 50 mL of aqueous suspension of 0.5 mol/L calcium hydroxide was prepared. 50 mL of 0.3 mol/L orthophosphoric acid was slowly added at 0.5 mL/minute rate into calcium hydroxide suspension. Then, 2 gram ciprofloxacin was added to this and pH (10.5) was carefully adjusted by 1 mol/L ammonium hydroxide. The suspension was well stirred (600 rpm) using magnetic stirrer for 30 minutes and aged overnight at room temperature. Precipitates were subjected to vacuum filtering using Büchner funnel, repeatedly washed with deionized water and filtered again. The precipitates were dried at room temperature for 48 hours. Dried lamps were ground by clean pestles and mortars.

### 2.3. Determination of Drug Concentration and Drug Loading

Filtrate of the suspension of HAp-ciprofloxacin composites, which were obtained after washing by deionized water, was taken and analyzed to determine the drug loading and the drug incorporation efficiency. Absorbance values of filtrates were measured at 274 nm using a UV-VIS spectrophotometer (Thermo Spectronic UV-1, USA). Thus, drug concentration and drug loading were calculated [14]:

(1) $$\begin{array}{cc} & \text{drug}\;\text{concentration}\;\left(\text{\%}\text{w}/\text{w}\right)\\ & \;\;=\frac{\text{drug}\;\text{incorporated}}{\text{net}\;\text{dry}\;\text{powder}\;\text{mass}\;\text{after}\;\text{loading}}\times 100,\\ & \text{drug}\;\text{loading}\;\left(\text{\%}\text{w}/\text{w}\right)\\ & \;\;=\frac{\text{drug}\;\text{incorporated}}{\text{drug}\;\text{added}\;\text{in}\;\text{the}\;\text{synthesis}\;\text{process}}\times 100. \end{array}$$

### 2.4. Manufacturing of Implants

HAp-ciprofloxacin composites (2 gram) were mixed with 2.5 mL of 1.5% (w/v) aqueous solution of guar gum to make a smooth paste and then poured into 2.5 mm diameter cylindrical moulds (diameter 2.5 mm) by using extruder syringe and dried at room temperature for 24 hours. After drying, the rods were removed from moulds and cut into cylindrical implants (2 × 2.5 mm) [14].

### 2.5. *In Vivo * Ciprofloxacin Release Study

#### 2.5.1. Animals

Adult male New Zealand white rabbits with weighing range of 2000–2500 grams were used for the *in vivo* evaluation of HAp-ciprofloxacin implants. Before surgery, the rabbits were acclimatized to their new environment and fed standard laboratory diet. The rabbits were caged individually with a constant temperature. The G.C.T.S. Animal Ethical Committee, Gupta College of Technological Sciences, Asanosl-1, India approved the study protocol.

#### 2.5.2. Study Protocol

5 Hap-ciprofloxacin implants were used for implantation. Before implantation, implants were sterilized by soaking ethyl alcohol. The applied model was designed to stimulate the local antibiotic release from implants after implantation in rabbit bone. Standard surgical technique was applied, including premedication, anesthesia, and surgical preparations. Rabbits were anaesthetized with 75 mg ketamine hydrochloride/kg body weight. A cortical bone window was created in the right proximal tibia of each rabbit. The bone marrow was removed with saline lavage, and 5 implants (total avg. wt., 40 mg) were implanted into the window created on the right proximal tibia. After surgery, functional activity was not limited and the animals received standard postoperative pain medication. The rabbits were euthanized at 3 days and 6 days. Serum and bone marrow concentrations of ciprofloxacin were determined from the harvested tibia.

#### 2.5.3. Determination of Ciprofloxacin in Bone and Serum after Implantation

High performance liquid chromatography (HPLC) analysis was carried out using a UV-detector (L-7400) and Winchrom software. The column used for separation of ciprofloxacin was a LiChro CART 250-4 LiChrosorb RP-18 (4.6 mm i.d.) column. The mobile phase consisted of 13 volume of acetonitrile R and 87 volumes 2.45 gram/L solution of phosphoric acid R and then adjusted to pH, 3 with triethylamine. All reagents used for the preparation of mobile phase were of HPLC grade. The flow rate of mobile phase was 1.5 mL/minute and the wavelength was 278 nm.

The bone marrow specimens were homogenized with a homogenizer, and finely homogenized bone marrow specimens was weighed and mixed with 5 mL of water. This suspension was centrifuged at 3000 rpm for 10 minutes. 0.5 mL of the supernatant of this suspension was mixed with 1 mL of acetonitrile by shaking for 15 minutes. Then this mixture was centrifuged at 2000 rpm for 10 minutes. The supernatant liquid evaporated, and the residue was mixed with 5 mL of mobile phase and further centrifuged at 3000 rpm for 10 minutes. The supernatant was filtered with a 0.45 *μ*m membrane filter. Finally, 50 *μ*L of sample was injected into the HPLC column.

0.5 mL of serum sample was mixed with 1 mL acetonitrile by shaking for 15 minutes. Then this mixture was centrifuged at 2000 rpm for 10 minutes. The supernatant liquid evaporated and the residue was mixed with 5 mL of mobile phase and again centrifuged at 3000 rpm for 10 minutes. The supernatant was filtered with a 0.45 *μ*m membrane filter, and 50 *μ*L of sample was injected into the HPLC column. 

Standard samples, control test samples were handled identically.

#### 2.5.4. Determination of *In Vivo* Ciprofloxacin Release from Implants Collected from Harvested Tibia after Implantation

*In vivo* ciprofloxacin released from the implanted HAp-ciprofloxacin implants after collection of harvested tibia was determined by the difference of amount of ciprofloxacin measured from 5 unused composites and 5 implanted composites collected from harvested tibia.

Implants were powdered and mixed with 10 mL of 0.1 (N) HCl solutions separately. After sonication of 20 minutes, these two suspensions were filtered. 0.5 mL of filtrates was mixed with 1 mL acetonitrile by shaking for 15 minutes and was mixed with 5 mL of mobile phase. The solution was filtered with a 0.45 *μ*m membrane filter, and 50 *μ*L of sample was injected into the HPLC column.

## 3. Results and Discussion

HAp-ciprofloxacin composites were synthesized by precipitation technique using these following parameters: 2 grams of ciprofloxacin added in the synthesis process, 600 rpm of stirring speed, and 0.5 mL/minute of orthophosphoric acid addition rate in the synthesis process. The drug concentration and drug loading in the synthesized HAp-ciprofloxacin composites were determined as 64.10 ± 0.60 and 86.60 ± 5.70% w/w, respectively (Table 1).

**Figure 1:**
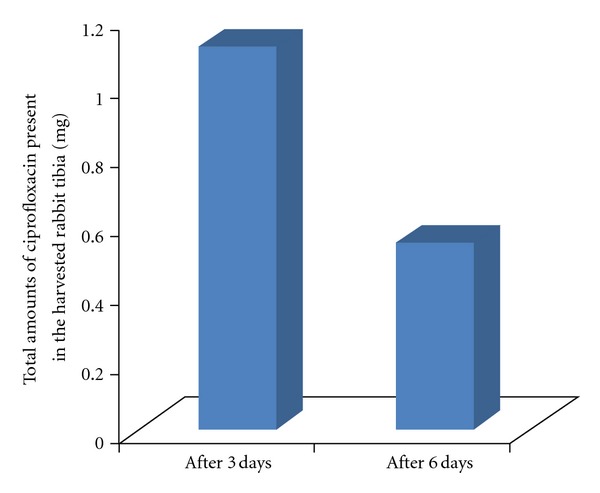
Total amount of ciprofloxacin (mg) present in the harvested rabbit tibia after implantation of HAp-ciprofloxacin bone implants.

After 3 days of implantation, the ciprofloxacin amount in the harvested tibia was 1.11 ± 0.03 mg. The total amount of ciprofloxacin present in harvested tibia after 6 days of implantation was 0.54 ± 0.02 mg (Figure 1). This result indicated high local antibiotic concentration at implantable site of the bone. 

**Figure 2:**
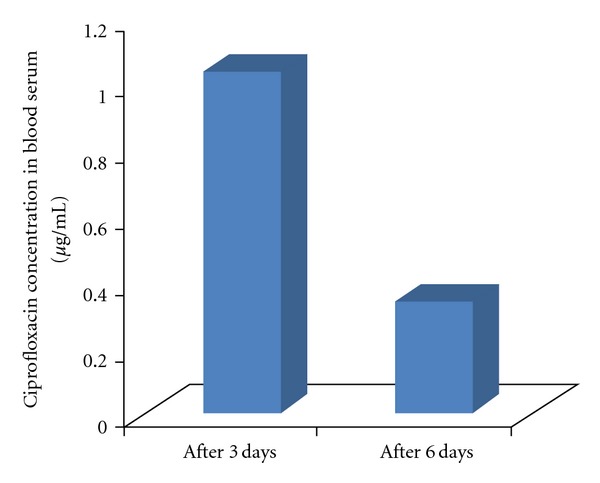
Ciprofloxacin concentration in blood serum (*μ*g/mL) after implantation of HAp-ciprofloxacin bone implants in rabbit tibia.

The ciprofloxacin concentration in blood serum after 3 and 6 days was presented in Figure 2. The concentration of ciprofloxacin in blood serum remained extremely low (1.03 ± 0.04 and 0.34 ± 0.01 *μ*g/mL after 3 days and 6 days of implantation, resp.) which indicates the remarkable limitation of systemic exposure. This can be useful to overcome the problems of serious side effects related to the systemic administration of antibiotics for a long period.

**Figure 3:**
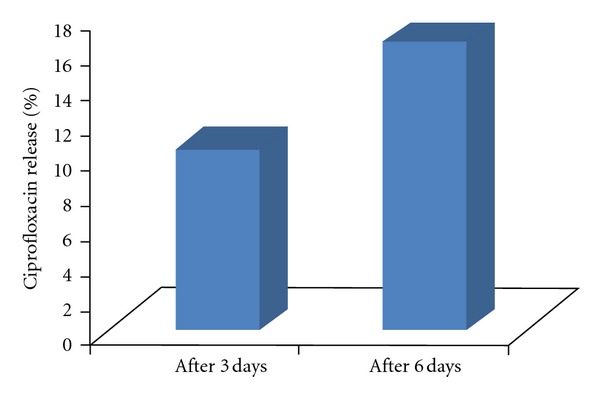
Percentage of *in vivo *ciprofloxacin release in the rabbit tibia measured from implanted implants after collection from the harvested tibia.

The percentage of* in vivo *ciprofloxacin release from implants collected from harvested tibia after implantation at 3 and 6 days interval was calculated and presented in Figure 3. After 3 days of implantation, 10.18% ciprofloxacin was released from the implants; whereas this was 12.75% after 6 days of implantation. The results of *in vivo *ciprofloxacin measurement from the HAp-ciprofloxacin implants showed 1.54 ± 0.05 mg after 3 days of implantation, which was quite greater than the results obtained from the harvested tibia after same days of interval (1.11 ± 0.03 mg). This difference could be attributed due to some amounts of ciprofloxacin may enter into the systemic circulation or may be utilized at the implant site. Also, there are a number of factors, such as perfusion, and bioresorbability of HAp in the bone, which may enhance the drug release from HAp-based implants.

## 4. Conclusion

This study is the preliminary investigation of the suitability of *in vivo* ciprofloxacin release from HAp-ciprofloxacin bone implants in rabbit tibia, after implantation, and these implants can be useful for the treatment of bacterial bone infections like osteomyelitis. The results of the study displayed the availability of elevated local antibiotic concentration at the implanted site with the limitation of systemic antibiotic exposure, which can be useful to minimize the risk of systemic toxicity-related side effects.

## Figures and Tables

**Table 1:** Drug concentration and drug loading in the synthesized HAp-ciprofloxacin composites by precipitation technique.

Synthesis parameters			Drug concentration^a^	Drug loading^a^
Ciprofloxacin	Stirring speed	Orthophosphoric acid addition rate		
2 grams	600 rpm	0.5 mL/minute	64.10 ± 0.60% w/w	86.60 ± 5.70% w/w

^a^Mean ± Standard Deviation, *n* = 3.
